# Resection of a Catecholamine-Elaborating Retroperitoneal Paraganglioma Invading the Inferior Vena Cava

**DOI:** 10.1155/2014/837054

**Published:** 2014-12-28

**Authors:** E. M. Mannina, Z. Xiong, R. Self, E. Kandil

**Affiliations:** ^1^Department of Radiation Oncology, Indiana University SOM, 535 Barnhill Drive, RT-041, Indianapolis, IN 46202, USA; ^2^Department of Pathology, Tulane University SOM, 1430 Tulane Avenue, SL-79, New Orleans, LA 70112, USA; ^3^Department of Surgery, Tulane University SOM, 1430 Tulane Avenue, SL-22, New Orleans, LA 70112, USA

## Abstract

Paragangliomas are rare tumors originating outside of the adrenal medulla which can be associated with catecholamine secretion or mass effect, one of which typically leads to their discovery. The differences between these tumors and traditional intra-adrenal pheochromocytomas are a subject of recent investigations. Standard of care therapy is medical management and surgical resection of the tumor. When tumors are biochemically active, medical optimization of the autonomic nervous system is a critical component to a safe, definitive resection. Tumors arising in the retroperitoneum present technical challenges for the surgeon as they are often large and difficult to access, making an oncologic resection much more difficult. Lastly, these tumors are mostly benign and rarely invade adjacent structures—an operative finding not always predicted by preoperative imaging—which, if present, adds significant complexity and risk to the resection. A case illustrating these challenges in the management of a biochemically active retroperitoneal paraganglioma invading the inferior vena cava follows.

## 1. Introduction

Pheochromocytomas arise from chromaffin cells of the adrenal medulla. They are often discovered clinically due to their elaboration of catecholamines leading to a predictable symptomatology of tachycardia and/or palpations, hypertension, sweating, and headaches. Cells of similar embryologic origin include those of the sympathetic and parasympathetic ganglia, the carotid and aortic bodies, and the organs of Zuckerkandl. Tumors arising from these extra-adrenal sites are denoted “paragangliomas” and the distinction from pheochromocytomas (intra-adrenal sympathetic paragangliomas) is receiving due interest in the literature regarding differential genetic predisposition, biochemical activity, metastatic potential, and prognosis. Paragangliomas do possess the capacity to release catecholamines similar to pheochromocytomas, often prompting their discovery. These tumors may arise in a wide array of locations and thus may present with diverse symptoms of mass effect. Rare retroperitoneal origins may result in tumors growing very large, potentially compressing or even invading other organs before discovery. One such case is presented here.

## 2. Case Report

**Figure 1:**
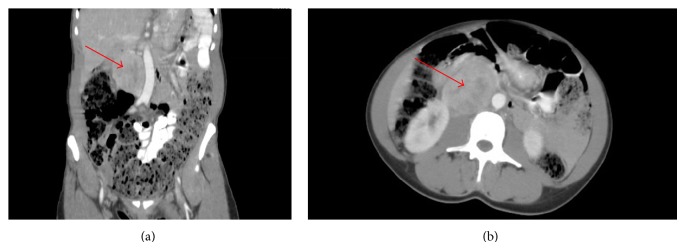
Paraganglioma (red arrow) visualized on computed tomography scan with IV contrast in coronal (a) and axial (b) planes without definitive evidence of local invasion.

**Figure 2:**
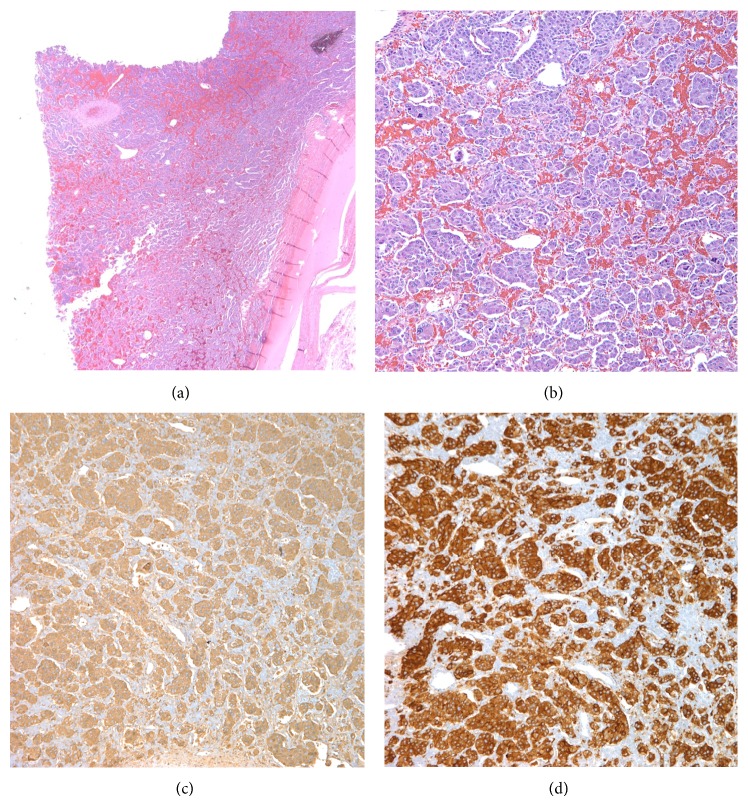
Paraganglioma demonstrated with hematoxylin and eosin staining at 2x magnification (a) and 10x magnification (b) revealing characteristic well-defined “Zellballen” nest pattern of tumor cells bound by a delicate fibrovascular stroma. At 10x magnification, immunohistochemical staining is positive for chromogranin (c) and synaptophysin (d). Images courtesy of Tulane University School of Medicine, Department of Pathology.

**Figure 3:**
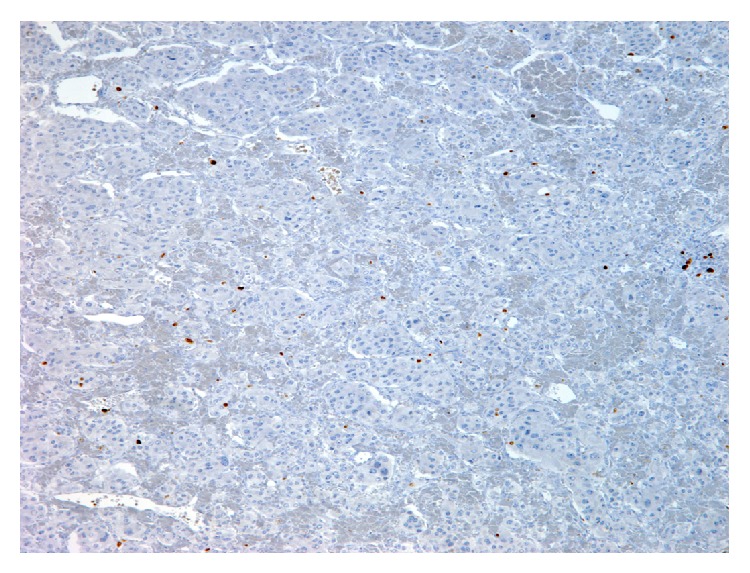
Paraganglioma shown at 10x magnification with Ki-67 staining demonstrating a low proliferative index. Image courtesy of Tulane University School of Medicine, Department of Pathology.

A 45-year-old African-American female presented with chest pain and shortness of breath associated with nausea, vomiting, headache, and diaphoresis. Admit vital signs were consistent with hypertension which was being treated with amlodipine and metoprolol. Two 24-hour urine collections demonstrated vanillylmandelic acid 64, 56.6 (N: 2–7 mcg/24 hr); normetanephrine 8364, 7406 (N: 75–375 mcg/24 hr); norepinephrine 1857, 1644 (N: 15–80 mcg/24 hr). CT scan of the abdomen revealed a moderately enhancing, homogeneous retroperitoneal mass measuring 5.9 × 4.9 cm with significant mass effect on neighboring structures and vasculature without appreciable invasion (Figure 1). A I-131 metaiodobenzylguanidine (MIBG) scan showed uptake at the abdominal mass only. CT-guided biopsy revealed cells with salt-and-pepper chromatin, abundant eosinophilic cytoplasm with a granular appearance, and classic Zellballen nest pattern (Figures 2(a) and 2(b)). Immunohistochemical staining demonstrated positivity for chromogranin (Figure 2(c)) and synaptophysin (Figure 2(d)) with a low Ki-67 proliferative index (Figure 3). A diagnosis of retroperitoneal paraganglioma (extra-adrenal pheochromocytoma) was declared. Patient was started on alpha blockade with prazosin.

**Figure 4:**
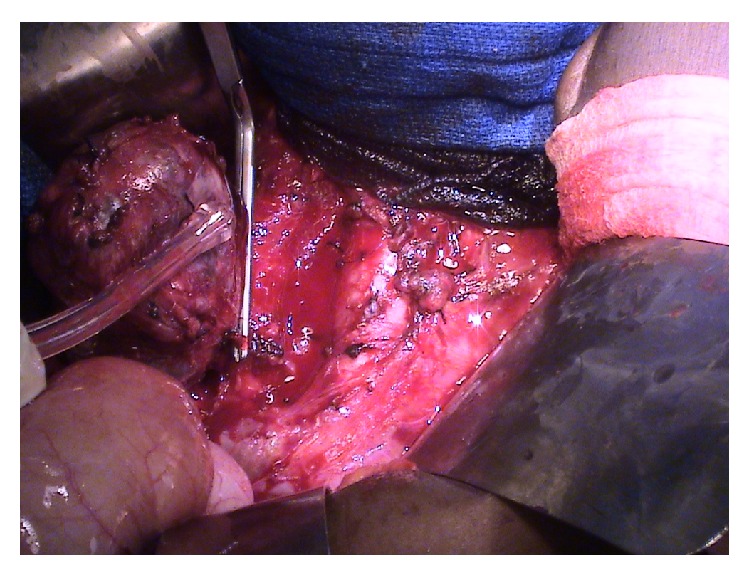
Intraoperative photograph of paraganglioma retracted to reveal vascular clamp about the underlying inferior vena cava. Image courtesy of Tulane University School of Medicine, Department of Endocrine Surgery.

**Figure 5:**
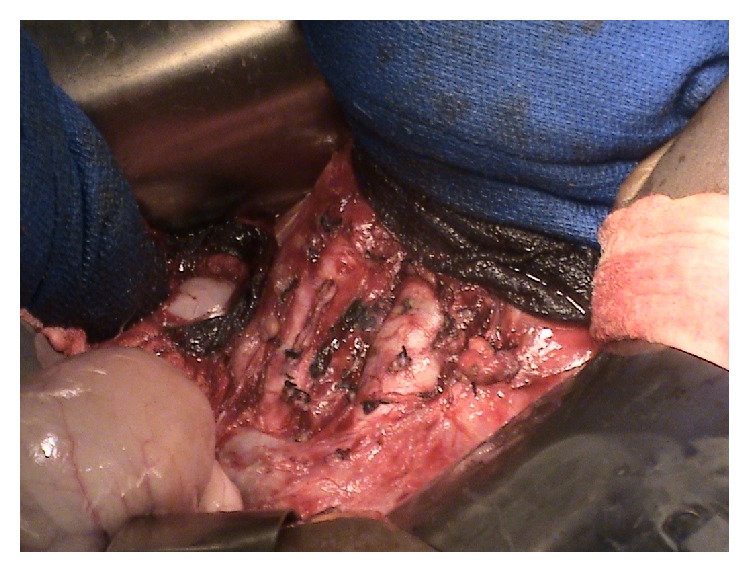
Intraoperative photograph demonstrating reconstructed inferior vena cava status after en bloc resection of attached paraganglioma. Image courtesy of Tulane University School of Medicine, Department of Endocrine Surgery.

Days prior to her surgical appointment, patient was admitted for hypertensive urgency with a blood pressure of 202/157 secondary to medication noncompliance due to nausea. Blood pressure was controlled with combined alpha and beta blockade; then patient was cleared for surgery. Upon entering the abdomen, impressive dilated bowel was noted secondary to mass compression. A dissection plane between the retroperitoneal mass and the transverse colon, duodenum, pancreas, and right kidney was achieved. Because the mass could not be separated from the inferior vena cava (IVC), a vascular clamp was applied (Figure 4) and the junction was resected en bloc. Four draining vessels were ligated and immediately the patient converted from stable hypertensive to hypotensive. The anesthesia team restored normotension. The IVC was then reconstructed (Figure 5). Feeding arteries were ligated and the mass was lifted from the aorta and freed. Several periaortic lymph nodes were sampled, bowel obstruction was ruled out, and the abdomen was closed. Surgical pathology revealed a 7 cm paraganglioma with IVC invasion, negative margins, and a single reactive lymph node. As of last follow-up, patient remained asymptomatic, normotensive, and without evidence of disease.

## 3. Conclusion

A 2001 retrospective review of 236 cases of benign paraganglioma by the Mayo Clinic provided data on these rare neoplasms finding that the majority (69%) of these tumors occurred in the head and neck. Just 21.5% were abdominal and of those 38% were periaortic/pericaval, 20% were perirenal, and 18% were at the organ of Zuckerkandl. All patients with periaortic or pericaval masses who were screened for catecholamine excess were discovered to have hyperfunctioning tumors, 95% resulting in hypertension [1]. A 2010 review of 67 retroperitoneal paraganglioma cases found similarly that 57% were periaortic (without further specification) and 82% were hyperfunctional [2]. Our case supports the data of periaortic paragangliomas typically being hyperfunctional.

A retrospective review from Johns Hopkins focused on retroperitoneal paragangliomas which comprised 22 of 253 (8.7%) paraganglioma resections. They documented average size (7.4 cm), rate of metastasis (9.5%), and rate of biochemical functionality (57.1%) [3]. Five-year overall survival after resection was 73% with prognosis significantly worse after metastasis, the rates of which were independent of tumor diameter, functionality, surgical margin status, and lymph node involvement [3]. 41% of tumors required adjacent organ resection, best predicted by a tumor size greater than 7 cm [3]; our case supports this conclusion. A 1990 study from Memorial Sloan Kettering found complete resection to be associated with 75% 5-year overall survival rate in retroperitoneal paragangliomas, which in their study had a rate of metastasis of 50% [4]. It has been documented that neither histology nor local invasion can predict malignant potential in these tumors, instead leaving metastasis as the sole method of confirmation [4, 5].

Accepted treatment for retroperitoneal paragangliomas is resection with annual follow-up biochemical testing [6], though this is limited by only a fraction of tumors being biochemically functional [1–3]. Retroperitoneal tumors often present with mass effects as discovered in our patient who had several segments of dilated bowel due to compression by the tumor. An additional complication is local invasion into vascular structures such as the aorta or inferior vena cava. Few cases exist in the literature of successful en bloc resections of retroperitoneal paragangliomas that invaded major vessels [7, 8]. One such case noted tight adhesion of the tumor to the aorta and inferior vena cava [8] while another suggested possible origin from the wall of the inferior vena cava [7], the latter ruled out by surgical pathology in our case. En bloc resection of many tumor types requiring vascular reconstruction has been documented with vascular patency rates at 24 mos. of 90% [9] with acceptable levels of morbidity and mortality confirmed in other studies [10]. Partial inferior vena cava resection with reconstruction was achieved in our patient and, as of last follow-up, vascular patency remained preserved.
